# The Moderating Effect of Cultural Orientation on the Relationship Between Growth Mindset and Learning Self-Efficacy: A Dimension-Specific Pattern

**DOI:** 10.3390/bs14121155

**Published:** 2024-12-02

**Authors:** Wu-Jing He, Kai Zhang

**Affiliations:** Department of Special Education and Counselling, The Education University of Hong Kong, 10 Lo Ping Road, Tai Po, New Territories, Hong Kong SAR, China

**Keywords:** growth mindset, cultural orientation, learning self-efficacy, moderating effect

## Abstract

Building on the theoretical perspectives of mindset theory and cultural orientation framework, this study explores the moderating role of cultural orientation in the relationship between individuals’ growth mindset and learning self-efficacy, addressing the contextual dependency of mindset theory. A total of 307 Chinese undergraduates (61% female; age range = 18–22 years) from a university in Hong Kong were recruited for this cross-sectional study. Cultural orientation, growth mindset, and learning self-efficacy were assessed via the Chinese version of the Cultural Values Scale (CVScale), Growth Mindset Inventory (GMI), and Learning Self-Efficacy Scale (LSES), respectively. Linear multiple regression analysis was conducted to examine the hypothesized moderation effects. The results revealed an interesting dimension-specific pattern among the five dimensions of cultural orientation. While one dimension (i.e., long-term orientation) had a positive moderating effect on the relationship between growth mindset and learning self-efficacy, two dimensions (i.e., power distance and uncertainty avoidance) had negative moderating effects on that relationship. The two remaining cultural dimensions (i.e., individualism/collectivism and motivation towards achievement and success) did not exhibit any significant moderating effects. These findings underscore the interplay between a growth mindset, cultural orientation, and learning self-efficacy, emphasizing the influence of cultural factors on the outcomes of mindset interventions. This study highlights the need for culturally tailored educational practices and interventions to maximize the effectiveness of growth mindset theories in diverse contexts.

## 1. Introduction

Intelligence mindset (or implicit theory of intelligence), defined as individuals’ beliefs in their intellectual abilities in relation to the potential for growth [1], has gained increasing attention in psychological and educational research, particularly for its potential effect on educational success [2]. Theoretically, a growth mindset (or the incremental theory of intelligence), known as the belief that intellectual abilities are changeable, malleable, and developable with effort over time, is posited to facilitate positive learning outcomes [3,4]. Empirically, however, research on the growth mindset effect has yielded mixed results, with some studies supporting a positive growth mindset effect but others not [5]. Further empirical scrutiny is thus needed to develop a full understanding of the link between mindset and learning. In line with this research, the current study has two objectives. First, while most previous studies have focused on learning outcomes via performance-oriented constructs (e.g., test scores and school grades) [6], the present study addresses the effect of a growth mindset on a process-oriented construct—learning self-efficacy—which is understood as a motivational construct that concerns an individual’s self-belief in his or her ability to achieve desired learning outcomes [7]. In social cognitive theory [8], learning self-efficacy is positively linked to learning outcomes through its ability to facilitate the learning process of initiating and sustaining learning motivation [9]. Second, to echo the evolving trend in the field that highlights the context-dependent nature of a growth mindset [10], which emphasizes the essence of understanding the mindset effect within the broader cultural and ecological landscape [11], the current study accounts for cultural influence in uncovering the effect of a growth mindset on learning self-efficacy. In particular, the moderating effect of cultural orientation on the mindset–efficacy link is explored.

### 1.1. Growth Mindset and Learning Self-Efficacy

The implicit theory of intelligence (or mindset theory) [1] posits a positive link between a growth mindset and learning self-efficacy. According to this theoretical perspective, people with a growth mindset are known as incremental theorists who view intellectual abilities as not fixed but rather incremental over time with effort. Growth-minded individuals believe that intellect can be cultivated and nurtured through persistency, hard work, and effective strategies [3]. Regarding learning-related outcomes, this theoretical perspective suggests that students with a growth mindset believe that their academic performance is the result of the effort they make and the effectiveness of the strategies they use. They believe in the promising relationship between increased effort and increased learning and tend to be oriented towards a learning goal [4,12]. Focusing specifically on the connection between a growth mindset and learning self-efficacy, Kim and Karr [6] argued that students with a growth mindset have a greater sense of self-control over their academic success and, in turn, feel more confident in their ability to apply self-regulated learning strategies, thereby enhancing their learning self-efficacy.

In this context, mindset theory is in alignment with many theories of motivation in postulating a positive link between a growth mindset and learning self-efficacy. For example, the attribution theory of motivation [13] predicts that students with a growth mindset tend to attribute their academic success to internal variables (e.g., personal attitudes, acquired knowledge and skills, dedication, and hard work), which is linked to a stronger sense of responsibility for self-improvement via the achievement of learning outcomes. Such students tend to have greater motivation for academic engagement and a greater sense of self-efficacy in their ability to achieve educational success [14]. Similarly, the self-determination theory of motivation [15] postulates that students with a growth mindset tend to believe in their autonomy and self-determination with regard to academic outcomes. Such students have a greater sense of self-control and self-regulation in the learning process. They feel more autonomous and competent in managing their learning effort, which is linked to learning outcomes and in turn bolsters their self-efficacy in learning [16].

Indeed, several studies have offered initial empirical support for a positive link between a growth mindset and learning self-efficacy. For example, in a sample of 660 undergraduate students in the U.S., Kim and Karr [6] reported that a growth mindset of intelligence was associated with increased academic self-efficacy, assessed via the Course/Academic subscale of the College Self-Efficacy Instrument (CSEI) [17]. This scale focuses on measuring students’ self-confidence in managing different tasks related to academic coursework. Similarly, in a sample of 1536 secondary school students in mainland China, Zhao et al. [2] reported that a growth mindset of intelligence was associated with increased academic self-efficacy, assessed via the Chinese version of the Motivated Strategies for Learning Questionnaire (MSLQ) [18]. This scale focuses on assessing students’ motivational beliefs and learning strategies in self-regulated learning. Collectively, these findings support mindset theory regarding the positive relationship between students’ growth mindsets and self-efficacy in various aspects of learning, such as the management of academic tasks and self-regulated learning.

To expand this line of research, the first objective of the present study is to examine the generalizability of the findings regarding the mindset–efficacy link by investigating the predictive power of a growth mindset for learning self-efficacy via the use of an alternative sample (i.e., Chinese undergraduates in Hong Kong) and the application of an alternative measure (i.e., the Chinese version of the General Self-Efficacy Scale/GSES) [19]. The GSES is adapted to assess students’ self-efficacy specifically in terms of learning in this study. Drawing on the theoretical perspective of Dweck’s mindset theory [1] and relevant research findings, we formulated Hypothesis 1 as follows: Growth mindset is a positive predictor of learning self-efficacy.

### 1.2. The Moderating Effect of Cultural Orientation

To understand the mindset effect on potential outcomes, mindset theory [1] also highlights the importance of accounting for the contextual influence in which the mindset effect is considered [20]. In retrospect, mindset theory was built in light of Bandura’s [21] triadic reciprocal determination model under the framework of social cognitive theory, which highlights the dynamic and interdependent relationships between contextual and personal factors in influencing performance outcomes [8]. This theoretical perspective emphasizes that a growth mindset does not function alone in contributing to potential outcomes; rather, its effect is contextually dependent: supportive contexts strengthen the effect, whereas nonsupportive contexts weaken the effect [4]. Recently, Yeager and Dweck [11] proposed the mindset-plus-supportive-context framework to examine the essence of the mindset effect through a dynamic approach by accounting for the interplay between growth mindset and contextual factors. This approach offers a useful lens for understanding the inconsistent findings related to learning outcomes linked to a growth mindset [10].

Taking the cultural context into consideration, a growing body of empirical research has revealed the influence of culture on the effectiveness of a growth mindset in influencing educational outcomes. For example, Rissanen et al. [22] reported that Finnish elementary school teachers who adopted growth mindset pedagogy adjusted their approaches to align with local cultural values in relation to education. King and Trinidad [12] noted that in cultures emphasizing personal achievement, the impact of a growth mindset is more pronounced. More recently, Lou and Li [23] reported that growth mindset interventions yield positive results in cultures with supportive norms, whereas in cultures with more fixed-mindset orientations, the effects are minimal or even counterproductive. Additionally, Zhang and He [10] reported that individuals from a cultural context that highlights a long-term orientation are more inclined to adopt a growth mindset. In a meta-analysis, Bardach et al. [24] reported that the association between teachers’ growth mindsets and self-efficacy varies across cultures. In another meta-analysis, Sisk et al. [25] reported that the effectiveness of growth mindset interventions varies across cultural backgrounds, particularly among students who face challenges, such as cultural stereotypes about intelligence. Collectively, these findings underscore the importance of considering cultural influence when evaluating the effectiveness of a growth mindset on learning self-efficacy.

Hence, the second objective of the current study is to examine how cultural influence moderates the relationship between a growth mindset and learning self-efficacy. Specifically, Hofstede et al.’s [26] cultural orientation framework is applied to support a systematic and nuanced examination of the moderating effect according to five cultural orientation dimensions: (1) power distance; (2) uncertainty avoidance; (3) individualism/collectivism; (4) motivation towards achievement and success; and (5) long-term/short-term orientation. This cultural orientation framework is the most widely used framework in cultural studies in psychological and educational research [27]. The paragraphs below highlight the key features of the five cultural orientation dimensions, with a focus on their possible impact on the relationship between a growth mindset and learning self-efficacy in educational settings.

First, power distance refers to the degree of acceptance of unequally distributed power [26]. In cultures that value high power distance, educational environments are typically hierarchical, with clear authority figures and limited student autonomy [28]. Empirical evidence suggests that high power distance in classrooms tends to limit student engagement and autonomy, which is essential for cultivating a growth mindset and enhancing self-efficacy [29]. Empirical evidence also suggests that students in high power distance cultures tend to report lower school belongingness, which is closely linked to learning self-efficacy, due to the hierarchical and generally unsupportive nature of these environments [30]. These findings suggest that high power distance can lead to hierarchical, rigid, and unsupportive school cultures that may inhibit the openness and autonomy necessary for a growth mindset. Hence, a negative moderating effect of power distance on the impact of a growth mindset on learning self-efficacy is expected.

Second, uncertainty avoidance refers to the degree to which students are comfortable with ambiguity and uncertainty in educational contexts [26]. A cultural orientation that emphasizes high uncertainty avoidance suggests a preference for strict rules, structured learning environments, and standardized assessments, which can discourage risk-taking and flexibility [31]. Empirical evidence suggests that high uncertainty avoidance in educational settings tends to lead to more rigid and less exploratory learning behaviors, potentially undermining the cultivation of learning self-efficacy [32]. Research has also revealed that high uncertainty avoidance weakens the effects of school leadership on instructional practices by limiting teachers’ collective efficacy [33]. This suggests that similar constraints could apply to students’ development of a growth mindset. These findings collectively suggest that a cultural orientation with high uncertainty avoidance may lead to rigid adherence to rules and an aversion to ambiguity, which may hinder the development of a growth mindset and its positive effects on learning self-efficacy. Hence, uncertainty avoidance is expected to have a negative moderating effect on the impact of a growth mindset on learning self-efficacy.

Third, the individualism/collectivism dimension captures the extent to which students prioritize personal goals over group objectives within educational settings [26]. A culture orientation that values individualism is more closely linked to the concern about the rights of each individual. Individualistic cultural orientation places a strong emphasis on personal achievements and self-reliance. In contrast, collectivist culture orientation values group harmony, cohesion, and shared successes [34]. Interestingly, despite these differences, research findings indicate that the relationship between a growth mindset and self-efficacy is not significantly moderated by whether a culture leans towards individualism or collectivism. Zhang [35] reported that both Chinese (more collectivist) and Finnish (more individualist) students held growth mindsets associated with academic success, suggesting that the relationship between a growth mindset and self-efficacy operates similarly across these culturally distinct groups. Arieli and Sagiv [36] also reported that this cultural dimension does not necessarily influence the effect of a growth mindset on personal development. These findings suggest that the effect of a growth mindset on learning self-efficacy is consistent across the cultural orientations of individualism and collectivism. Hence, the individualism–collectivism cultural dimension is expected to have a null moderating effect on the mindset–efficacy link.

Fourth, motivation towards achievement and success, formerly referred to as masculinity/femininity, reflects the cultural orientation for either competitive or cooperative learning atmospheres among students [26]. This terminology was recently updated by Hofstede Insights [37] to better align with contemporary perspectives on gender and to address concerns about treating gender as a binary concept. The new term, motivation towards achievement and success, shifts the focus from gendered connotations to cultural preferences for competitiveness and achievement versus cooperation and empathy, reflecting a more inclusive and modern approach to cultural analysis. A cultural orientation emphasizing motivation towards achievement prioritizes competitiveness and individual accomplishments, fostering environments that value personal excellence and goal-oriented behaviors. In contrast, an orientation leaning towards success through collaboration highlights cooperation, empathy, and support, creating settings where collective well-being and harmony are prioritized. Despite these distinctions, research suggests that the relationship between a growth mindset and self-efficacy is not significantly moderated by whether a culture leans towards masculinity or femininity. For example, Zhang [35] reported that cultural orientations along this dimension do not significantly predict the formation of growth mindsets among teachers. Similarly, Li and Burkholder [38] found that orientations toward achievement and success do not alter the impact of a growth mindset on self-efficacy in educational settings. These findings suggest that the effectiveness of a growth mindset in enhancing learning self-efficacy remains consistent, regardless of differences along this cultural dimension. Hence, motivation towards achievement and success is expected to present a null moderating effect on the mindset–efficacy relationship.

Finally, long-term/short-term orientation refers to a cultural dimension that indicates the extent to which people prioritize future-focused values (long-term orientation) versus immediate gratification (short-term orientation) [26]. A long-term orientation refers to the fostering of virtues oriented towards future rewards, particularly perseverance and thrift. Its opposite pole, short-term orientation, stands for the fostering of virtues related to fulfilling immediate needs [26]. Some characteristics of long-term orientation include persistence, lack of emphasis on leisure time, saving, and being thrifty, whereas short-term orientation emphasizes quick results, leisure time importance, and spending. Cultures with a long-term orientation value delayed gratification, dedication, hard work, lifelong-skill development, and adaptability, which align well with the principles of a growth mindset. In contrast, short-term-oriented cultures focus more on quick academic success and immediate gratification [39]. In this line, Figlio et al. [40] reported that students from long-term-oriented cultures show a stronger commitment to learning and achieve better academic outcomes. Additionally, Miller and Xu [41] demonstrated that individuals with long-term orientations are more likely to engage in behaviors that prioritize sustained growth and development. These findings underscore the compatibility between long-term orientation and growth mindset principles, suggesting that fostering a long-term perspective in educational settings may enhance the impact of a growth mindset on learning self-efficacy.

In summary, drawing on the theoretical perspectives of Dweck’s [1] mindset theory and Hofstede et al.’s [26] cultural orientation framework, as well as relevant research findings, we hypothesize (Hypothesis 2) that there exists a domain-specific moderating effect of cultural orientation on the relationship between a growth mindset and learning self-efficacy. More specifically, power distance (Hypothesis 2a) and uncertainty avoidance (Hypothesis 2b) have negative moderating effects on the mindset–efficacy relationship, whereas individualism/collectivism (Hypothesis 2c) and motivation towards achievement and success (Hypothesis 2d) have null moderating effects, and long-term orientation (Hypothesis 2e) has a positive moderating effect.

## 2. Methods

### 2.1. Participants and Procedures

This study employed a cross-sectional design to examine the moderating role of cultural orientation in the relationship between growth mindset and learning self-efficacy. An initial sample of 320 undergraduate students from a university in Hong Kong was recruited for this study. Following data cleaning, which led to the exclusion of 13 participants due to incomplete responses (attrition rate = 4.06%), the final sample consisted of 307 students (61% female; *M*_age_ = 20.27 years, *SD* = 4.03, range = 18–22 years), all of whom were ethnic Chinese. The participants had an average of 13.5 years of education (*SD* = 1.02; range = 13–15 years) and included a mix of 37% (*n* = 114) first-year students, 21% (*n* = 64) second-year students, 17% (*n* = 52) third-year students, and 25% (*n* = 77) fourth-year students. Prior to participation, all potential participants who expressed interest in joining the study were informed of the study objectives, confidentiality protocols, and voluntary nature of their involvement. Informed consent was obtained from each participant before data collection commenced. Data were collected via a combination of digital questionnaires administered via the Qualtrics and Sojump platforms, as well as traditional paper-based surveys, allowing flexibility in response formats. Assessments of the study variables (i.e., growth mindset, cultural orientation, and learning self-efficacy) were conducted via standardized instructions in classroom settings, with groups of 30–40 students per session. The assessment process took approximately 25–30 min to complete. To address potential fatigue and order effects, the sequence of tasks was counterbalanced across participants, with any potential biases distributed evenly to ensure that no specific order influenced the outcomes. This approach aligns with best practices for mitigating order effects in psychological research [42]. All procedures adhered to ethical guidelines set by the Human Research Ethics Committee of the affiliated institution, ensuring participant confidentiality, data integrity, and adherence to voluntary participation standards.

### 2.2. Instruments

#### 2.2.1. Growth Mindset

Growth mindset was measured via the Chinese adapted version of the growth mindset subscale, the Growth Mindset Inventory (GMI) [1], which assesses individuals’ mindset beliefs about the malleability of intelligence. This 4-item scale is one of the most widely used growth mindset scales in mindset belief research [43]. An example statement from the scale is “Even your basic intelligence level can be increased considerably.” Participants responded to the statements on a 6-point rating scale ranging from 1 = strongly disagree to 6 = strongly agree, with a higher score indicating a greater degree of endorsement that intelligence is malleable. More specifically, a scoring system developed by Claro et al. [44] was applied to classify participants’ mindset beliefs, where scores from 1 to 2 suggest a fixed mindset, scores from 2.1 to 4.9 suggest a mixed mindset, and scores from 5 to 6 suggest a growth mindset. Previous research has supported the internal consistency of the scale with Cronbach’s α = 0.76 − 0.88 and its structural validity with an exploratory factor analysis, which yielded a communality range of 0.59 to 0.72 and a factor loading range of 0.77 to 0.85 [45]. The applicability of the scale in a Chinese school context has also been supported for both teacher [10] and student samples [46]. In this study, the scale also demonstrated high internal consistency, with Cronbach’s α = 0.79. Furthermore, the structural validity of the scale was supported by the results of a confirmatory factor analysis (CFA) with acceptable fit indices (χ^2^/*df* = 2.45; RMSEA = 0.05; AGFI = 0.92; GFI = 0.93; IFI = 0.96; CFI = 0.96). These findings confirm the reliability and validity of the Growth Mindset Inventory in assessing mindset beliefs in this study.

#### 2.2.2. Cultural Orientation

Cultural orientation was assessed via the Chinese adapted version of the Individual Cultural Values Scale (CVSCALE) [47], which is a 26-item five-dimensional scale of individual cultural values that assesses participants’ cultural orientations under the following themes: (1) power distance (e.g., “People in higher positions should make most decisions without consulting people in lower positions”); (2) uncertainty avoidance (e.g., “Instructions for operations are important”); (3) collectivism/individualism (e.g., “Individuals should sacrifice self-interest for the group”); (4) motivation towards achievement and success (e.g., “It is more important for men to have a professional career than it is for women”); and (5) long-term/short-term orientation (e.g., “Giving up today’s fun for success in the future”). The participants rated their levels of agreement with the items on a five-point scale ranging from 1 = strongly disagree to 5 = strongly agree. Higher scores indicate that individuals have high power distance and uncertainty avoidance, a collectivist structure, masculine attributes, and long-term oriented focus. Yoo et al. [47] reported satisfactory average reliability ranging from 0.72 to 0.78 across cultural dimensions and offered supporting evidence for construct validity with acceptable fit indices of CFA (χ^2^/*df*: 2.30; RMSEA = 0.071; CFI = 0.94; NFI = 0.90). Many subsequent studies have confirmed the adequate reliability and validity of the CVSCALE across diverse cultural contexts [48,49], including a Chinese cultural context [50]. In the present study, we also obtained good Cronbach’s alpha values (α = 0.81). Furthermore, the results of the CFA indicated that the scale had an acceptable fit for construct validity: χ^2^/*df* = 2.67; RMSEA = 0.04; AGFI = 0.91; GFI = 0.92; IFI = 0.95; CFI = 0.95. These results demonstrate the CVSCALE’s reliability and validity in measuring cultural orientations in the present study.

#### 2.2.3. Learning Self-Efficacy

Learning self-efficacy was assessed via the Chinese version of the general self-efficacy scale [19]. This 10-item scale was adapted to focus on measuring participants’ self-efficacy in relation to learning. Example items include “I can solve most problems in learning if I invest the necessary effort” and “If I am in trouble in learning, I can usually think of something to do”. The participants responded to the statements on a 4-point rating scale ranging from 1 = strongly disagree to 4 = strongly agree, with a higher score indicating greater learning self-efficacy or a greater degree of self-confidence with respect to learning. Previous studies have supported the psychometric property of measuring students’ academic self-efficacy in a learning context, with adequate internal consistency, i.e., Cronbach’s α = 0.65−0.84 and good fix indices of the CFA, i.e., CFI = 0.96; TLI = 0.94; RMSEA = 0.078; SRMR = 0.038 [51]. Previous studies have also supported the applicability of the scale in a Chinese educational context, with acceptable Cronbach’s α = 0.86−0.91 and fit indices of the CFA (RMSEA = 0.06; CFI = 0.99; TLI = 0.97; SRMR = 0.05) [52]. In the present study, the internal consistency of the scale was 0.82. The results of the CFA demonstrated a satisfactory fit for the construct validity of the scale (χ*^2^*/*df* = 2.35; RMSEA = 0.05; AGFI = 0.93; GFI = 0.95; IFI = 0.96; CFI = 0.96). This supports the reliability and validity of the learning self-efficacy scale in evaluating academic self-efficacy in this study.

## 3. Data Analysis

Linear multiple regression analysis was conducted to examine the moderating effects of cultural orientation dimensions on the relationship between growth mindset and learning self-efficacy. First, Pearson correlation analysis was performed to examine the bivariate correlations among the study variables (i.e., growth mindset belief, cultural orientation, and learning self-efficacy), with *r* ≥ 0.10 (≤−0.10) indicating a small effect; *r* ≥ 0.30 (≤−0.30) indicating a medium effect; and *r* ≥ 0.50 (≤−0.50) indicating a large effect [53]. To test the hypotheses, linear multiple regression analyses were performed to reveal the main effect of a growth mindset, as shown in Model 2, and the moderating effect, as shown in Model 3, controlling for the possible covariate effects of demographic variables (i.e., age, gender, and education) in Model 1. Standardized regression coefficients (i.e., β statistics) were used to understand the strength of the main effect of the predictor variable (i.e., growth mindset belief) on the outcome variable (i.e., learning self-efficacy); the larger the value, the stronger the predictive effect. The moderation effects of various dimensions of cultural orientation were examined by using the interaction term “cultural orientation × growth mindset belief”. Simple slope plots were subsequently used to illustrate the distinct manifestations of the outcome variable (i.e., learning self-efficacy) under high (*M* + 1 *SD*) and low (*M* − 1 *SD*) levels of the hypothesized moderating variables (i.e., cultural orientations). This graphical illustration was developed with reference to a common practice used in similar research [54] to showcase the effect of a moderating variable on the relationship between the predictor and outcome variables. In all analyses, a *p* value of less than 0.05 was regarded as indicating statistical significance.

## 4. Results

### 4.1. Bivariate Correlations

Table 1 displays the means, standard deviations, reliability estimates, and intercorrelations of the study variables. In line with Hypothesis 1, the *r* statistics illustrate that the hypothesized predictor variable, mindset, was positively correlated with the outcome variable, learning self-efficacy (*r* = 0.53; *p* = 0.001). With respect to its correlation with the hypothesized moderating variable, cultural orientation, a dimension-specific pattern was observed. In line with Hypotheses 2a−2b, a growth mindset was significantly negatively correlated with power distance (*r* = −0.42; *p* = 0.005) and uncertainty avoidance (*r* = −0.46; *p* = 0.005). In line with Hypotheses 2c−2d, growth mindset showed no significant correlation with collectivism (*r* = 0.04; *p* = 0.11) or motivation towards achievement and success (*r* = 0.07; *p* = 0.09). In line with Hypothesis 2e, growth mindset showed a significant and positive correlation with long-term orientation (*r* = 0.47; *p* = 0.005). Regarding the relationship between the hypothesized moderating variable (i.e., cultural orientation) and the outcome variable (i.e., learning self-efficacy), the *r* statistics also suggest a domain-specific pattern. Specifically, in line with Hypotheses 2a−2b, learning self-efficacy was significantly negatively correlated with power distance (*r* = −0.20; *p* = 0.008) and uncertainty avoidance (*r* = −0.22; *p* = 0.008). In line with Hypotheses 2c−2d, learning self-efficacy was not significantly correlated with collectivism (*r* = 0.08; *p* = 0.13) or motivation towards achievement and success (r = 0.06; *p* = 0.14). In line with Hypothesis 2e, learning self-efficacy was significantly positively correlated with long-term orientation (*r* = 0.21; *p* = 0.008).

### 4.2. Regression Analyses

**Figure 1:**
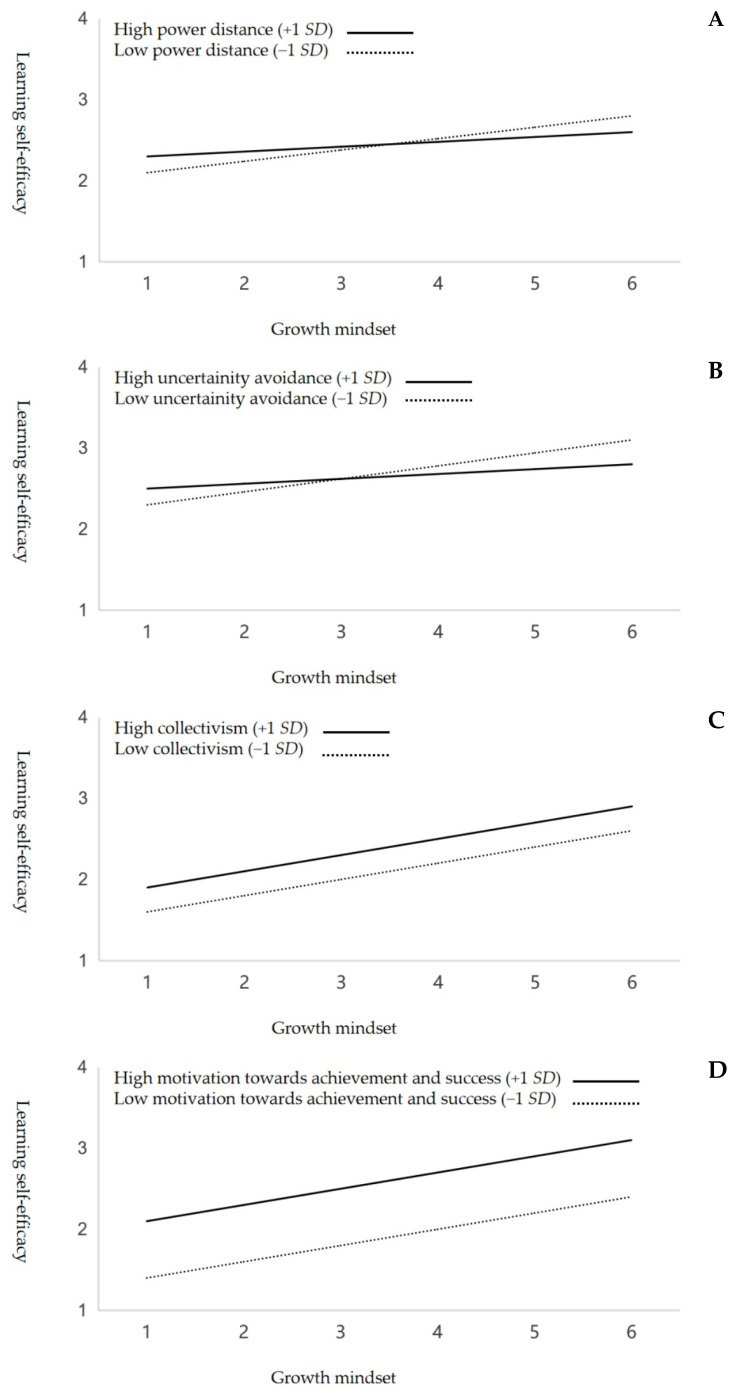

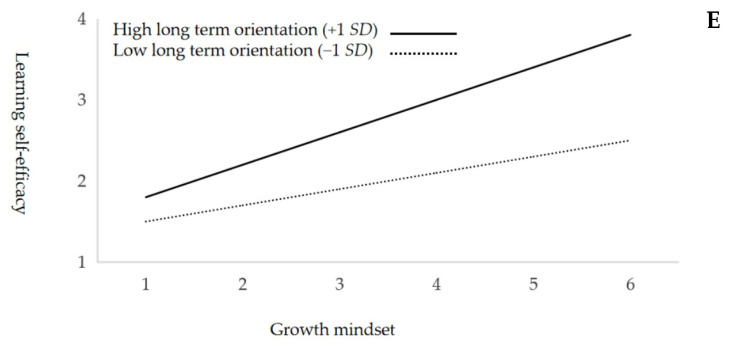
Cultural orientation showed a pattern of dimension-specific moderating effects: power distance (**A**) and uncertainty avoidance (**B**) had negative moderating effects; collectivism/individualism (**C**) and motivation towards achievement and success (**D**) had null moderating effects; and long-term orientation (**E**) had a positive moderating effect.

Table 2 displays the results of the regression analyses with respect to the testing of the hypotheses, and Figure 1 depicts a graphical representation of these results. As posited in Hypothesis 1, the results of Model 2 illustrate that growth mindset was positively predictive of learning self-efficacy (β = 0.28; *p* < 0.001) and explained 11% of the variance in learning self-efficacy (Δ*R*^2^ = 0.11; Δ*F* = 23.23; *p* < 0.001) when the effect of demographic variables in Model 1 was controlled (*R*^2^ = 0.02; *F* = 4.13; *p* = 0.17). With respect to Hypothesis 2, which anticipates a domain-specific moderation effect of cultural orientation on the relationship between growth mindset and learning self-efficacy, the statistics of model change, as shown in Model 3, revealed a significant increase in explanatory power (Δ*R*^2^ = 0.14; Δ*F* = 27.18; *p* < 0.001) when the interaction terms of growth mindset × cultural orientation were introduced into the model, in which context the overall model fit was statistically significant (*F* = 58.61; *p* < 0.001). Specifically, power distance (β = −0.11; *p* = 0.008) and uncertainty avoidance (β = −0.13; *p* = 0.006) demonstrated a negative moderation effect on the relationship between growth mindset and learning self-efficacy (see also Panels A and B of Figure 1), which supported Hypotheses 2a–2b. Individualism/collectivism (β = 0.05; *p* = 0.13) and motivation towards achievement and success (β = 0.03; *p* = 0.14) had no significant moderation effect on this link (see also Panels C and D of Figure 1), which aligned with Hypotheses 2c–2d. Finally, long-term orientation (β = 0.14; *p* = 0.008) had a positive and significant moderation effect on this relationship (see also Panel E of Figure 1), supporting Hypothesis 2e.

## 5. Discussion

This study explored the moderating role of cultural orientation in the relationship between a growth mindset and learning self-efficacy among Chinese university students in Hong Kong. The findings revealed several key insights. First, a positive correlation was established between a growth mindset and learning self-efficacy, reinforcing the theoretical underpinnings of growth mindset theory. Second, a specific cultural dimension—long-term orientation—positively moderated the relationship between a growth mindset and learning self-efficacy, suggesting that a forward-looking, perseverance-focused cultural perspective enhances the benefits of a growth mindset on learning-related outcomes. In contrast, other cultural dimensions, such as power distance and uncertainty avoidance, were found to negatively moderate this relationship, indicating that hierarchical structures and a preference for certainty can undermine the effectiveness of growth mindset interventions. For instance, in cultures with higher power distance, educational environments might emphasize hierarchy and limited student autonomy [30], potentially constraining the openness and self-directed learning behaviors that support the effective application of a growth mindset [55]. In contrast, lower power distance may provide a more collaborative and autonomous learning environment [30], which could better align with growth mindset principles [43]. Similarly, cultures with high uncertainty avoidance, with their stronger focus on structured learning and strict rules [56], might reduce opportunities for risk-taking and exploration, which are often associated with growth mindset strategies [57]. By comparison, lower uncertainty avoidance may offer a more flexible and exploratory context [56], so it could be more conducive to the application of growth mindset beliefs [58]. Nevertheless, other cultural dimensions, such as individualism/collectivism and motivation towards achievement and success, did not have any moderating effects on the mindset–efficacy relationship. The mindset–efficacy relationship was consistently stronger among students with a collectivistic cultural orientation than among those with an individualistic orientation. Similarly, the mindset–efficacy relationship was consistently stronger among students with a cultural orientation valuing motivation towards achievement and success. These findings underscore the importance of considering cultural factors in the application and adaptation of growth mindset interventions, suggesting that cultural sensitivity is crucial for optimizing educational outcomes through mindset theories.

### 5.1. Theoretical Implications

The findings of this study underscore the influence of cultural orientation on the relationship between a growth mindset and learning self-efficacy, demonstrating a dimension-specific pattern. Building upon the existing body of research [2,6], this investigation not only solidifies the positive link between a growth mindset and learning self-efficacy but also delves into the moderating influences of cultural orientation. Specifically, it highlights the positive moderating role of long-term orientation, which strengthens the effect of a growth mindset on learning self-efficacy. This effect, as hinted at by Hofstede et al., [39], suggests that individuals with a long-term outlook are more likely to postpone immediate satisfaction, tackle complacency and exert extra effort in pursuit of long-term goals. These behaviors align closely with the core principles of a growth mindset, which emphasizes resilience, persistence, and long-term development. This alignment between long-term orientation and the principles of a growth mindset amplifies learning self-efficacy, illustrating how a future-focused outlook can enhance the effectiveness of a growth mindset. Conversely, the study revealed that power distance and uncertainty avoidance had negative moderating effects, indicating that these cultural dimensions significantly weaken the impact of a growth mindset on learning self-efficacy. These cultural dimensions, which emphasize strict control and regulation, can overshadow individual autonomy and flexibility, which are crucial for the development of a growth mindset [26]. In such settings, the effectiveness of a growth mindset in promoting self-efficacy may be limited, as these cultural orientations create barriers that challenge the openness and adaptability required for a growth mindset to flourish. Interestingly, the study finds no significant moderating impacts within the frameworks of collectivism and motivation towards achievement and success. In the case of collectivism, the consistent benefits of a growth mindset—such as increased perseverance and resilience—may stem from underlying cultural values that transcend the focus on individual or collective achievements. However, this influence remains unclear, and further explanation is needed to fully understand its dynamics. Similarly, for masculinity, the willingness to address challenges and learn from setbacks—a core tenet of a growth mindset—may be attributed to a cultural emphasis on competitiveness and individual achievements, which fosters environments that value personal excellence. Nonetheless, this influence warrants further exploration to clarify the specific mechanisms at play.

Collectively, these findings highlight the complexity and specificity of cultural moderating effects on the relationship between a growth mindset and self-efficacy. They provide valuable insights into the future development of growth mindset theory, suggesting that integrating the mindset-plus-supportive-context framework [11] could be crucial for understanding how mindsets interact with contextual factors, such as cultural dimensions. This approach emphasizes that mindset interventions cannot be isolated from contextual influence (e.g., cultural influence); instead, they must be tailored to align with local values and structures. Furthermore, this dimension-specific pattern offers a nuanced understanding of how particular cultural values—such as long-term orientation, power distance, and uncertainty avoidance—moderate the growth mindset–self-efficacy link. For example, previous studies have shown that long-term orientation promotes perseverance and resilience, which aligns well with growth mindset principles, thereby enhancing self-efficacy [40]. Conversely, high power distance has been associated with reduced autonomy and limited student engagement, which can weaken the benefits of a growth mindset [30]. Similarly, uncertainty avoidance has been linked to a preference for rigid rules and structured learning environments, potentially limiting the flexibility required for growth mindset development [32]. These findings collectively support the observed moderating effects of cultural dimensions in this study. Compared with previous studies, such as Rissanen et al. [59], who demonstrated support for growth mindset formation through local inclusive educational philosophies, and Zhang et al. [35], who illustrated varying feedback patterns across countries, this study adds depth by examining these cultural dimensions separately. The findings underscore the need for a culturally adaptable approach when implementing growth mindset interventions, ensuring that educational strategies are effectively tailored to fit the cultural landscape. This nuanced perspective not only enriches the current understanding of growth mindset theory but also provides a pathway for future research to explore how the mindset-plus-supportive-context framework can be operationalized in diverse cultural environments to maximize the effectiveness of growth mindset principles.

### 5.2. Practical Implications

The findings provide insights that help explain the unsuccessful replication of the effect of growth mindset theory on the prediction of learning outcomes in previous studies. For example, data analysis from the PISA 2018 indicates that China and Lebanon are the only two countries among 74 developing nations in which a growth mindset is negatively correlated with PISA scores, with the authors speculating that this is due to contextual heterogeneity and considering such “unexplained heterogeneity should be a starting point for future theory development” ([4], p. 1273). The findings of this study imply that the influence of mindsets on learning-related outcomes is not direct but rather moderated by cultural factors; however, more empirical research is needed to consolidate this conclusion, as only learning self-efficacy has been investigated in relation to cultural factors. In essence, the failure to account for contextual factors when implementing growth mindset interventions may be responsible for the lack of replicability in certain contexts, thereby contributing to the unexplained heterogeneity in the effectiveness of the growth mindset. In a more recent study, Burnette et al. [60] reviewed growth mindset interventions from 2002, the year of the first mindset intervention, through the end of 2020 and revealed that the field requires a deeper comprehension of the optimal contexts for these interventions, with attention to the specific cultures or environments that create settings conducive for growth and development. The findings of the present study reinforce this conclusion and further suggest that because of the moderating effect of cultural orientation, efforts to implement a growth mindset in one cultural setting may not translate directly to another setting. These findings highlight the importance of adaptability and cultural sensitivity in designing and implementing further growth mindset interventions. Despite the single-sample design focused on a university in Hong Kong, the findings demonstrate the relevance of cross-cultural frameworks, such as Hofstede’s cultural dimensions, in understanding how cultural values moderate the growth mindset’s impact on learning self-efficacy. Hong Kong’s unique blend of Eastern and Western cultural influences offers an opportunity to study these dimensions within a context characterized by both high power distance and high uncertainty avoidance. These findings contribute to understanding cultural dimensions that may also apply to other regions with similar cultural characteristics.

Consequently, the present study argues that the growth mindset framework might be oversimplified, especially when it is implemented in a real context. From the perspective of growth mindset theory, individuals are divided into incremental theorists and entity theorists on the basis of their beliefs about intelligence and distinguished through their behaviors [3]. However, the cultural moderating effects revealed in the present study remain unexplained within this framework. This oversight may result in a partial understanding—or even misinterpretation—of how the growth mindset operates across different cultural contexts. It risks overlooking the profound influence of cultural values on individuals’ beliefs about intelligence and their corresponding behaviors. Practically, the findings offer guidance for educational practices in settings with similar cultural characteristics. For example, in classrooms with high power distance, enhancing student autonomy and reducing hierarchical barriers could strengthen the effects of growth mindset interventions. Similarly, in high-uncertainty-avoidance contexts, adopting more flexible and exploratory teaching methods may better support the application of growth mindset strategies. These recommendations highlight the potential for culturally informed interventions to optimize the benefits of growth mindset in diverse educational environments. Furthermore, such oversight could lead to misinterpretations of the effectiveness of growth mindset interventions, as failures or successes could be attributed solely to individual differences without consideration of the significant role of the cultural context. In summary, the present study suggests the necessity of integrating cultural factors into the delivery of growth mindset interventions for further implementation.

### 5.3. Limitations and Recommendations for Future Studies

Several limitations of the study should be noted in the interpretation of its results. First, the participants were all undergraduates from a single university in Hong Kong, such that students at various developmental stages or from other geographical areas were not represented. This sampling limitation may restrict the diversity of cultural orientations, potentially limiting the observed effects of certain dimensions, such as individualism/collectivism, which may exhibit greater variation across broader cultural contexts. To strengthen the generalizability and robustness of the findings, future research should endeavor to include students of diverse geographical, economic, and cultural backgrounds. Second, the limitations of this study’s cross-sectional design prohibit definitive causal inferences, despite the theoretical and empirical evidence presented. Particularly in research contexts such as this, where complex multivariable relationships are under examination, future research aiming to explain causality would significantly benefit from employing experimental, prospective, or longitudinal methodologies.

Third, a notable limitation of this study is the scope of the learning outcomes measured, which was confined primarily to learning self-efficacy. While learning self-efficacy is a critical component of learning-related outcomes, the influence of a growth mindset extends beyond this single dimension to encompass aspects such as motivation, engagement, and helplessness. The exclusive focus on self-efficacy in learning thus narrows the lens through which the broader effects of a growth mindset on learning outcomes can be understood. In addition, the moderating effects on the relationships between a growth mindset and these learning-related outcomes are not solely restricted to cultural orientation. Other significant factors, such as school climate and policy guidance, could also play crucial roles in shaping this dynamic. Future research should aim to incorporate a broader array of variables to more comprehensively explore the mechanisms through which a growth mindset impacts learning outcomes. The inclusion of these additional variables could not only provide a richer understanding of the multifaceted nature of a growth mindset but also facilitate the development of more nuanced, contextually sensitive applications of growth mindset theory. Such an expansion of the research focus is essential for advancing the localization and practical implementation of growth mindset principles in diverse educational settings.

Fourth, we gathered data via self-reports, such that the scope of the field investigation in this study was limited. The pragmatic implementation of mindset beliefs by students in the real world remains largely unexplored. Future research could more deeply explore the actual expression of a growth mindset within authentic learning contexts. Essential avenues for further research include investigating whether Chinese students with a growth mindset demonstrate more discernible ability-oriented behaviors in their learning than their peers with fixed mindsets do and unravelling the underlying reasons behind any observed variations. Moreover, it is critical to ascertain whether students’ interpretations of growth mindset behaviors align with Dweck’s [1] original conceptualization of growth mindset theory. This is particularly pertinent in light of research [61] highlighting discrepancies in the manifestation of mindset beliefs across different contexts, suggesting that the expression of the same belief could vary significantly, depending on contextual factors.

## 6. Conclusions

In conclusion, this study contributes to the understanding of the relationship between a growth mindset and cultural orientation by highlighting the ways in which cultural factors moderate the relationship between students’ growth mindsets and learning outcomes. By examining the role of cultural orientation in Hong Kong, this research not only elucidates the mechanisms through which a growth mindset influences learning self-efficacy but also sheds light on the broader implications of applying growth mindset interventions across diverse cultural contexts. The findings underscore the critical need for integrating cultural sensitivity and adaptability into the design and implementation of educational interventions aimed at fostering growth mindsets. Moreover, this study calls attention to the unexplained heterogeneity in growth mindset research, suggesting that future investigations should delve deeper into the cultural dimensions that influence the efficacy of growth mindset interventions. As our understanding advances, it becomes increasingly clear that a one-size-fits-all approach to a growth mindset may not suffice. Instead, tailored strategies that account for the rich tapestry of cultural beliefs and values are imperative for maximizing the potential benefits of growth mindset principles in enhancing learning outcomes. This study paves the way for further exploration into how cultural differences can be effectively incorporated into educational practices, thereby enriching our understanding and supporting the implementation of growth mindset theory in a global educational landscape.

## Figures and Tables

**Table 1:** Descriptive statistics, reliability estimates, and intercorrelations of the study variables.

Variables	Mean (SD)	1	2	3	4	5	6	7	8	9	10
1. Age	20.3 (4.03)	-									
2. Gender	-	0.03	-								
3. Education	13.5 (1.02)	−0.01	0.03	-							
4. Growth mindset	5.27 (1.65)	0.09	−0.03	0.05	(0.91)						
5. Power distance	4.67 (1.02)	−0.09	−0.02	−0.05	−0.42 **	(0.88)					
6. Uncertainty avoidance	4.61 (0.98)	0.03	−0.06	0.03	−0.46 **	0.04	(0.86)				
7. Collectivism	4.21 (0.53)	−0.01	0.03	0.05	0.04	0.09	0.05	(0.81)			
8. Motivation towards achievement and success	3.77 (0.39)	0.06	−0.08	0.03	0.07	−0.06	−0.04	0.03	(0.82)		
9. Long-term orientation	4.89 (0.41)	0.05	0.02	0.03	0.47 **	−0.05	−0.03	−0.04	0.03	(0.85)	
10. Learning self-efficacy	3.22 (0.42)	0.07	0.05	0.05	0.53 ***	−0.20 *	−0.22 **	0.08	0.06	0.21 **	(0.83)

Note: *n* = 307. The diagonal values in parentheses represent the alpha-reliability coefficients. * *p* < 0.05; ** *p* < 0.01; *** *p* < 0.001.

**Table 2:** Results of regression analyses.

	Learning Self-Efficacy		
Independent Variables	M1	M2	M3
*Step 1*			
Age	0.01	0.01	0.01
Gender	0.02	0.02	0.02
Education	0.01	0.01	0.01
*Step 2*			
Growth mindset		0.28 ***	0.28 ***
*Step 3*			
Growth mindset × power distance			−0.11 **
Growth mindset × uncertainty avoidance			−0.13 **
Growth mindset × collectivism			0.05
Growth mindset × motivation towards achievement and success			0.04
Growth mindset × long term orientation			0.14 **
*R* ^2^	0.02	0.13 **	0.27 **
*F*	4.13	41.78 ***	58.61 ***
Δ*R*^2^	--	0.11 **	0.14 **
Δ*F*	--	23.23 ***	27.18 ***

Note: *n* = 307. ** *p* < 0.01; *** *p* < 0.001.

## Data Availability

The data presented in this study are available on request from the corresponding author due to privacy, legal, or ethical reasons.
